# Supplementary material for: Deletion of wfs1 Impairs Oligodendrocyte Precursor Cells Dorsal Distribution and Myelination Through the wfs1-hmgcs1 Axis in Zebrafish
Source: Biology (Basel). 2026 Aug 16;15(16):1402. doi: 10.3390/biology15161402 (PMC13510151; doi:10.3390/biology15161402)
Supplement: Supplementary file 1 [file biology-15-01402-s001.zip › File S1-Transcriptome sequencing data and differentially expressed genes (DEGs) in wfs1-- zebrafish at 5 dpf/2.KEGG/wfs1KOvsWT/GSEA/wfs1KO/GLUTATHIONE_METABOLISM/GLUTATHIONE_METABOLISM(DRE00480).html]

Details for gene set GLUTATHIONE\_METABOLISM(DRE00480)[GSEA]

|  || Dataset | deg.sample |
| Phenotype | sample.cls |
| Upregulated in class | wfs1KO |
| GeneSet | GLUTATHIONE\_METABOLISM(DRE00480) |
| Enrichment Score (ES) | 0.40892255 |
| Normalized Enrichment Score (NES) | 1.680425 |
| Nominal p-value | 0.0 |
| FDR q-value | 0.09457217 |
| FWER p-Value | 0.224 |
Table: GSEA Results Summary

  

Fig 1: Enrichment plot: GLUTATHIONE\_METABOLISM(DRE00480)      
 Profile of the Running ES Score & Positions of GeneSet Members on the Rank Ordered List

  

| PROBE | DESCRIPTION (from dataset) | GENE SYMBOL | GENE\_TITLE | RANK IN GENE LIST | RANK METRIC SCORE | RUNNING ES | CORE ENRICHMENT || 1 | 406762\_1 | pgd |  |  | 66 | 4.835 | 0.0754 | Yes |
| 2 | 100330864 | LOC100330864 |  |  | 70 | 4.818 | 0.1526 | Yes |
| 3 | 555317 | si:ch211-276a23.5 |  |  | 156 | 4.461 | 0.2214 | Yes |
| 4 | 570579\_1 | g6pd |  |  | 730 | 2.516 | 0.2438 | Yes |
| 5 | 450084\_1 | gss |  |  | 836 | 2.334 | 0.2779 | Yes |
| 6 | 100006589\_1 | idh1 |  |  | 876 | 2.275 | 0.3131 | Yes |
| 7 | 393493 | gstt2 |  |  | 1479 | 1.665 | 0.3209 | Yes |
| 8 | 431762\_1 | mgst1.2 |  |  | 1991 | 1.372 | 0.3268 | Yes |
| 9 | 393778 | prdx6 |  |  | 2148 | 1.307 | 0.3429 | Yes |
| 10 | 571365 | gstm.2 |  |  | 2205 | 1.283 | 0.3617 | Yes |
| 11 | 393191 | gpx8 |  |  | 2428 | 1.197 | 0.3739 | Yes |
| 12 | 569014 | ggt1b |  |  | 2440 | 1.194 | 0.3927 | Yes |
| 13 | 553575 | gsr |  |  | 2662 | 1.122 | 0.4037 | Yes |
| 14 | 101884069 | si:ch73-59p9.2 |  |  | 3023 | 1.030 | 0.4089 | Yes |
| 15 | 406278 | ggctb |  |  | 4161 | 0.800 | 0.3860 | No |
| 16 | 723997 | mgst2 |  |  | 4718 | 0.716 | 0.3800 | No |
| 17 | 447895 | gpx1b |  |  | 5104 | 0.666 | 0.3786 | No |
| 18 | 553169 | gstp2 |  |  | 5188 | 0.656 | 0.3865 | No |
| 19 | 794084 | si:ch73-111m19.2 |  |  | 5639 | 0.623 | 0.3823 | No |
| 20 | 563855 | chac1 |  |  | 6138 | 0.573 | 0.3759 | No |
| 21 | 100333757 | ggt5b |  |  | 6235 | 0.564 | 0.3819 | No |
| 22 | 368909 | rrm2b |  |  | 6644 | 0.524 | 0.3775 | No |
| 23 | 570579\_2 | g6pd |  |  | 6769 | 0.513 | 0.3818 | No |
| 24 | 570579 | g6pd |  |  | 7224 | 0.472 | 0.3751 | No |
| 25 | 326857 | gclc |  |  | 7445 | 0.457 | 0.3755 | No |
| 26 | 324900 | txndc12 |  |  | 7806 | 0.437 | 0.3712 | No |
| 27 | 100006589 | idh1 |  |  | 8531 | 0.401 | 0.3548 | No |
| 28 | 352926 | gpx1a |  |  | 9766 | 0.318 | 0.3211 | No |
| 29 | 449784\_1 | mgst1.1 |  |  | 9903 | 0.310 | 0.3218 | No |
| 30 | 352928 | gpx4a |  |  | 9915 | 0.309 | 0.3264 | No |
| 31 | 100002145 | si:ch73-337l15.2 |  |  | 9937 | 0.307 | 0.3307 | No |
| 32 | 100002956 | si:ch211-195e19.1 |  |  | 10032 | 0.301 | 0.3326 | No |
| 33 | 393387 | ggt1a |  |  | 10290 | 0.285 | 0.3291 | No |
| 34 | 322533 | anpepb |  |  | 10588 | 0.267 | 0.3240 | No |
| 35 | 406703\_1 | gsta.1 |  |  | 11380 | 0.223 | 0.3027 | No |
| 36 | 798788 | gpx3 |  |  | 11398 | 0.222 | 0.3057 | No |
| 37 | 494154 | lancl1 |  |  | 12582 | 0.160 | 0.2711 | No |
| 38 | 431762 | mgst1.2 |  |  | 12896 | 0.146 | 0.2636 | No |
| 39 | 100004652 | si:ch211-81n22.1 |  |  | 13002 | 0.140 | 0.2626 | No |
| 40 | 767682 | zgc:152830 |  |  | 13374 | 0.122 | 0.2528 | No |
| 41 | 563972\_1 | gstt1a |  |  | 13477 | 0.117 | 0.2515 | No |
| 42 | 30740 | rrm1 |  |  | 13918 | 0.096 | 0.2392 | No |
| 43 | 450084 | gss |  |  | 14972 | 0.048 | 0.2069 | No |
| 44 | 492500 | gsto2 |  |  | 15402 | 0.027 | 0.1938 | No |
| 45 | 562854 | lap3 |  |  | 16014 | -0.002 | 0.1746 | No |
| 46 | 563972 | gstt1a |  |  | 16711 | -0.023 | 0.1531 | No |
| 47 | 101882295 | ggt7 |  |  | 17094 | -0.036 | 0.1417 | No |
| 48 | 393556\_1 | gstt1b |  |  | 17163 | -0.039 | 0.1402 | No |
| 49 | 333974 | gclm |  |  | 17390 | -0.049 | 0.1339 | No |
| 50 | 564619 | gstr |  |  | 17772 | -0.066 | 0.1230 | No |
| 51 | 100148732 | ggt1l2.1 |  |  | 18065 | -0.079 | 0.1150 | No |
| 52 | 449784 | mgst1.1 |  |  | 18288 | -0.089 | 0.1095 | No |
| 53 | 567275 | mgst3b |  |  | 18399 | -0.094 | 0.1075 | No |
| 54 | 436894 | gsto1 |  |  | 18959 | -0.120 | 0.0919 | No |
| 55 | 100006589\_2 | idh1 |  |  | 19353 | -0.138 | 0.0817 | No |
| 56 | 114426 | odc1 |  |  | 19382 | -0.139 | 0.0831 | No |
| 57 | 795959 | si:dkey-222h21.12 |  |  | 19859 | -0.160 | 0.0707 | No |
| 58 | 552981 | gpx7 |  |  | 20037 | -0.168 | 0.0678 | No |
| 59 | 79381 | gstp1 |  |  | 20086 | -0.170 | 0.0690 | No |
| 60 | 393556 | gstt1b |  |  | 20106 | -0.171 | 0.0711 | No |
| 61 | 100004744 | LOC100004744 |  |  | 20726 | -0.200 | 0.0549 | No |
| 62 | 557614 | chac2 |  |  | 20898 | -0.209 | 0.0529 | No |
| 63 | 678541 | gstk4 |  |  | 21503 | -0.241 | 0.0377 | No |
| 64 | 80872 | sms |  |  | 21550 | -0.244 | 0.0402 | No |
| 65 | 406762 | pgd |  |  | 22903 | -0.320 | 0.0028 | No |
| 66 | 100334384 | gpx2 |  |  | 23238 | -0.343 | -0.0022 | No |
| 67 | 569734 | ggt5a |  |  | 23261 | -0.345 | 0.0027 | No |
| 68 | 386951 | idh2 |  |  | 23851 | -0.383 | -0.0097 | No |
| 69 | 100149204 | ggcta |  |  | 24620 | -0.423 | -0.0271 | No |
| 70 | 324366 | gstm.1 |  |  | 25161 | -0.457 | -0.0367 | No |
| 71 | 393493\_1 | gstt2 |  |  | 25358 | -0.472 | -0.0354 | No |
| 72 | 100151110 | oplah |  |  | 25631 | -0.496 | -0.0359 | No |
| 73 | 100002145\_1 | si:ch73-337l15.2 |  |  | 26810 | -0.610 | -0.0632 | No |
| 74 | 436833 | gstk1 |  |  | 27536 | -0.677 | -0.0751 | No |
| 75 | 352929 | gpx4b |  |  | 27634 | -0.691 | -0.0671 | No |
| 76 | 555478 | si:ch211-106j24.1 |  |  | 28214 | -0.774 | -0.0729 | No |
| 77 | 555478\_1 | si:ch211-106j24.1 |  |  | 28649 | -0.847 | -0.0730 | No |
| 78 | 393778\_1 | prdx6 |  |  | 29110 | -0.944 | -0.0723 | No |
| 79 | 406736 | mgst3a |  |  | 29458 | -1.048 | -0.0664 | No |
| 80 | 555317\_1 | si:ch211-276a23.5 |  |  | 29554 | -1.079 | -0.0521 | No |
| 81 | 568744 | gstm.3 |  |  | 29605 | -1.097 | -0.0361 | No |
| 82 | 30733 | rrm2 |  |  | 29935 | -1.219 | -0.0269 | No |
| 83 | 394009 | srm |  |  | 30543 | -1.536 | -0.0213 | No |
| 84 | 406703 | gsta.1 |  |  | 30735 | -1.713 | 0.0001 | No |
| 85 | 553575\_1 | gsr |  |  | 31216 | -2.255 | 0.0212 | No |
Table: GSEA details [plain text format]

  

Fig 2: GLUTATHIONE\_METABOLISM(DRE00480)      
 Blue-Pink O' Gram in the Space of the Analyzed GeneSet

  

Fig 3: GLUTATHIONE\_METABOLISM(DRE00480): Random ES distribution      
 Gene set null distribution of ES for **GLUTATHIONE\_METABOLISM(DRE00480)**
